# EUPopLink Country report - Iceland

**DOI:** 10.12688/openreseurope.23658.1

**Published:** 2026-05-07

**Authors:** Eiríkur BERGMANN

**Affiliations:** 1 Social Science, Bifröst University, Borgarnes, Western Region, 311, Iceland

**Keywords:** Euroscepticism, Iceland, Populism, European Integration, Sovereignty, Nationalism

## Abstract

This paper explores the unique nature and evolution of Euroscepticism in Iceland, which originates not from contemporary identity politics but from a deeply embedded national ideology prioritising sovereignty and democratic control over natural resources. Historically, Iceland’s relationship with Europe has been defined by a dual imperative: seeking economic prosperity through integration mechanisms like the European Economic Area, while fiercely protecting its hard-won political autonomy by resisting full European Union membership.

The study examines the multiparty political landscape, demonstrating how mainstream factions have traditionally navigated this ambivalence. Special emphasis is placed on the rise of populist and quasi-populist actors, notably the Centre Party and the People’s Party. The Centre Party deploys a hard, historically grounded Euroscepticism framing the EU as a threat to Icelandic self-determination, whereas the People’s Party utilizes a more reactive, welfare-centric critique.

However, the trajectory of Icelandic Euroscepticism is not static. The paper argues that recent geopolitical shocks – including the complexities of Brexit, the COVID-19 pandemic, the war in Ukraine, and shifts in U.S. foreign policy – have tempered nationalist resistance, prompting a pragmatic re-evaluation of external alignments. Consequently, a 2024 coalition government has committed to holding a referendum on resuming the EU accession process, signalling a potential paradigm shift in Iceland’s European orientation. Ultimately, this case study illuminates the complex interplay between nationalism, populism, and strategic pragmatism within a small-state setting.

## Plain Language summary

Icelanders have long hesitated to fully join the European Union, preferring to protect their hard-won independence and retain control over natural resources, particularly fisheries. To balance these concerns, Iceland cooperates economically with Europe through the European Economic Area (EEA) agreement but avoids the political oversight of full EU membership.

Populist factions, particularly the Centre Party and People’s Party, have capitalised on this national pride. They strongly oppose integration, warning that it would surrender Icelandic sovereignty to foreign elites and bureaucrats.

However, recent global shocks - including Brexit, the COVID-19 pandemic, and the war in Ukraine - have shifted this perspective. Recognising the need for collective security in a volatile world, a government coalition formed in late 2024 has pledged to hold a referendum on resuming EU accession talks.

## Introduction

Icelanders have long exhibited a distinctive form of political hesitation regarding deeper European integration. This ambivalence arises from a unique political culture, shaped by a strong sense of sovereignty developed through the country’s protracted struggle for independence from Denmark, and reinforced by its geographical remoteness and economic uniqueness. ^4^ At the same time, Iceland has shown a consistent commitment to participating in European integration institutions, driven by both practical economic considerations and a desire to establish its status as a fully sovereign, modern European state. ^10^ This dual imperative–protecting autonomy while seeking recognition and prosperity through cooperation – is central to Iceland’s relationship with the European Union, illuminating the evolution of its Euroscepticism. ^9^

On one hand, economic and strategic imperatives have drawn Iceland closer to European frameworks. As a small, open economy reliant on access to export markets, particularly in fisheries, energy, and tourism, Iceland has gradually become integrated into European structures. ^5^ It joined the European Free Trade Association (EFTA) in 1970 and became part of the European Economic Area (EEA) in 1994, thus securing access to the Single Market. In 2001, Iceland acceded to the Schengen Area, further aligning itself with the rest of continental Europe in terms of border management and mobility.

Conversely, profound political reservations persist. European integration is often depicted in Icelandic discourse as a possible threat to national sovereignty, particularly in sensitive areas such as fisheries policy, natural resource management, and legal autonomy. ^10^ This perspective is strengthened by a historical narrative in which Icelandic independence, achieved only in the mid-20th century, is still considered fragile and worthy of careful protection. ^14^ Formal membership in the EU, with its supranational legal and institutional framework, is consequently regarded by many Icelanders as a surrender of hard-won autonomy. ^3^

This ambivalence was strikingly revealed during and after the 2008 financial crisis. In the aftermath of the near-total collapse of its banking system, Iceland applied to join the European Union in July 2009 under the pro-European government led by the Social Democratic Alliance. For a fleeting moment, the EU was perceived by some as a means to stability, reform, and international support. ^5^ However, by 2013, the accession process had stalled, halted by a resurgence of nationalism and a growing Eurosceptic sentiment that portrayed Brussels as yet another external entity threatening Icelandic sovereignty. The Icesave dispute between the UK and the Netherlands, regarding the repayment of deposit guarantees, solidified this perception, igniting a backlash that contributed to the rise of nationalist and populist movements. ^7^

This report examines the development and transformation of Euroscepticism in Iceland. It argues that Icelandic Euroscepticism is not merely a product of economic interests or short-term political opportunism; rather, it reflects a deeply embedded national ideology in which sovereignty and modernity are not opposing poles but are seen as co-dependent. Within this framework, Euroscepticism can take various forms – from stringent nationalist resistance to soft pragmatic reticence – depending on the political actor and context. ^13^

Special attention is placed on the role of populist and quasi-populist actors in this landscape, particularly the Centre Party (Miðflokkurinn) and the People’s Party (Flokkur fólksins), which have employed Eurosceptic discourse to galvanise support and distinguish themselves from mainstream parties. ^6^ Simultaneously, recent geopolitical developments–including Brexit, the COVID-19 pandemic, Russia’s war in Ukraine, and the re-election of Donald Trump in 2024–have prompted a careful re-evaluation of Iceland’s external alignments. ^20^ Notably, a coalition government formed in late 2024, including the People’s Party, has committed to holding a referendum on whether to resume the EU accession process. This indicates a potential inflexion point in Iceland’s European orientation and introduces a new chapter in the ongoing narrative of Icelandic Euroscepticism. According to a survey by Prósent in early 2025, more respondents support Iceland’s membership of the European Union than oppose it, with 45% in favour, 35% against, and 20% neither for nor against. ^19^

The present country report was prepared as part of the EUPopLink COST Action ^1^ following the EUPopLink Theoretical Framework and guidelines. ^16^

## Iceland’s political landscape and party positions on Europe

The Icelandic party system is characterised by a multiparty configuration shaped by a proportional representation electoral system and a strong tradition of coalition government. ^22^ Unlike many European countries, attitudes towards European integration in Iceland do not align neatly along a left–right ideological axis. Instead, they are influenced by a blend of historical experiences, sectoral interests – particularly in fisheries and agriculture – and narratives of sovereignty, independence, and identity. ^2^

The Independence Party (Sjálfstæðisflokkurinn), a longstanding dominant force in Icelandic politics, epitomises the ambivalent stance many mainstream parties adopt towards European integration. While supportive of economic cooperation with Europe, particularly through the EEA, it remains opposed to full EU membership. ^17^ Its rhetoric often emphasises the protection of Icelandic sovereignty and control over natural resources, thereby allowing it to absorb Eurosceptic sentiment while retaining access to European markets. ^10^

The Progressive Party (Framsóknarflokkurinn), grounded in rural and agrarian interests, has historically maintained a cautious and often sceptical approach to European integration. ^6^ It has played a vital role in shaping the discourse regarding the significance of fisheries policy and the necessity of safeguarding Icelandic agriculture from EU regulation. However, following the departure of Sigmundur Davíð Gunnlaugsson and the establishment of the more hard-line Centre Party, the Progressive Party has adopted a more pragmatic stance, seeking to strike a balance between economic cooperation and national control.

On the other hand, the Social Democratic Alliance (Samfylkingin) stands out as traditionally the most consistently pro-European party in Iceland. Following the 2008 crash, it spearheaded the EU application process and has argued that EU membership would enhance Iceland’s global influence, modernise its regulatory frameworks, and provide economic stability. The Liberal Reform Party (Viðreisn), a relatively new centre-right formation that initially split from the Independence Party, also advocates for EU membership, viewing integration as a natural extension of Iceland’s liberal, open-market orientation.

The Left-Green Movement (Vinstri græn), out of parliament as of autumn 2024, has consistently opposed EU membership on the grounds of promoting Iceland’s sovereignty and emphasising decentralisation. Although not firmly anti-European, its scepticism often focuses on the EU’s perceived neoliberal tendencies and a lack of environmental ambition.

## Populist positions on ‘Europe’ and the Euroscepticism nexus

In this diverse landscape, the Centre Party and the People’s Party represent the most vocal expressions of contemporary Icelandic Euroscepticism. These parties have employed populist rhetoric, nationalist imagery, and anti-elite narratives to challenge Iceland’s engagement with Europe and to advocate for a more inward-looking, protectionist policy agenda. ^22^ Their rise has transformed the Eurosceptic discourse from a cautious mainstream stance to a more ideologically charged populist platform.

The Centre Party advocates a form of hard Euroscepticism. Since its inception, it has strongly opposed Iceland’s accession to the European Union. It has been openly critical of EU institutions, framing them as a threat to Icelandic autonomy and national interests. This opposition is not merely rhetorical but operational: the party mobilised extensively against the adoption of EU energy legislation in 2019, particularly its Third Energy Package, arguing that it would erode Iceland’s sovereignty over its energy sector. ^22^

Sigmundur Davíð Gunnlaugsson, the party’s founder and leader, portrayed the EU not just as a distant bureaucracy but as a force actively undermining Iceland’s self-determination. This framing positions the Centre Party as a nationalist bulwark against foreign encroachment, in continuity with historical narratives of independence and resistance.

The People’s Party, while also sceptical of EU integration, provides a critique that is more centred on welfare. Its Euroscepticism exists within a broader populist discourse that emphasises social justice for Icelandic citizens, particularly the elderly, the disabled, and the working poor. The EU is critiqued less for its institutional overreach and more for its association with immigration, neoliberalism, and misguided government priorities. Party leader Inga Sæland frequently asserts that Icelandic state resources are disproportionately allocated to foreigners at the expense of vulnerable Icelanders, and that international agreements – whether from Brussels or Geneva – undermine the state’s capacity to care for its own.

While both parties are clearly Eurosceptic, the Centre Party’s position is more ideologically consistent and historically grounded. It frequently references past conflicts, such as the Icesave dispute, to demonstrate what it perceives as foreign efforts to economically and politically subjugate Iceland. These narratives are rich in symbolic meaning, portraying Iceland as a small but proud nation continually compelled to defend its independence from powerful outsiders. The Centre Party’s Euroscepticism is, therefore, not merely issue-based but interwoven into its nationalist worldview.

The People’s Party, by contrast, displays a more reactive and context-dependent form of Euroscepticism. Its criticisms of European institutions are mainly strategic, aimed at supporting its claims that the political elite has become disconnected from ordinary people and that external forces are influencing Icelandic social policies. Its critique is arguably more opportunistic, aligning with specific populist grievances rather than presenting a fully formed ideological opposition to European integration. ^13^

### Impact of the national identity

Euroscepticism in Iceland is deeply rooted in the country’s nationalist tradition. Unlike many post-war European democracies, where nationalism was largely delegitimised after 1945, Iceland’s nationalism remained intact and central to its political identity. ^9^ Iceland emerged relatively unharmed from the Second World War and was still in the process of asserting its independence during the conflict. ^11^ Consequently, nationalism in Iceland retained a positive connotation, associated with self-determination, anti-colonialism, and cultural pride. ^15^

This historical continuity indicates that Euroscepticism in Iceland is more about safeguarding sovereignty and resisting external influence than about xenophobia or anti-immigrant sentiments (though such themes do appear in populist and radical right rhetoric). For the Centre Party and, to a lesser degree, the People’s Party, opposition to the EU naturally stems from this nationalist tradition. Their discourse is rich with references to the sagas, the Alþingi, the cod wars, and the Icesave dispute – all events that strengthen a national narrative of struggle and victory over external powers. ^3^

## Response to contemporary events

Recent global events have prompted a reconfiguration of Eurosceptic narratives in Iceland. While earlier periods were defined by nationalist resistance to integration, the current context reflects a more complex interplay between scepticism and strategic reconsideration.

The Brexit referendum and its aftermath initially emboldened Icelandic Eurosceptics. It was held up as proof that small nations could reclaim sovereignty from Brussels. Yet as the negative consequences of Brexit became more evident – economic uncertainty, political fragmentation, and diminished international influence – the narrative shifted. Rather than serving as a roadmap to follow, Brexit became a cautionary tale. Icelandic public discourse has increasingly emphasised the importance of maintaining stability through the EEA, while steering clear of the pitfalls of departing from European frameworks. ^22^

The COVID-19 pandemic further transformed the rhetorical landscape. Although the initial stages of the crisis saw a reassertion of national borders and a brief revival of self-reliance discourse, the broader experience demonstrated the limitations of small-state isolation. The EU’s coordinated vaccine procurement and distribution efforts, along with its collective economic stimulus, highlighted the potential benefits of deeper integration. In Iceland, this prompted more pragmatic discussions about Europe’s role in crisis management and the advantages of shared infrastructure. ^18^

The Ukraine war and Trump’s re-election in 2024 have similarly tempered Icelandic Euroscepticism, both within political parties and among the public. Russia’s invasion of Ukraine has underscored the importance of collective security and foreign policy coordination – areas where Iceland, as a small state, remains vulnerable. ^21^ Meanwhile, Trump’s return to power and his renewed scepticism towards NATO have raised concerns in Reykjavik about the dependability of traditional transatlantic alliances. These events have reignited discussions about the EU as more than just an economic partner, but also a geopolitical stabiliser in an increasingly volatile international landscape.

## Populist framing

Populist actors in Iceland consistently depict European integration as a betrayal by elites and a threat to democracy. ^6^ Sigmundur Davíð Gunnlaugsson, now leader of the Centre Party, in particular, has positioned himself as a defender of the Icelandic people against both domestic elites and foreign bureaucrats. His rhetorical style combines appeals to historical memory with contemporary grievances, suggesting that the political class is complicit in sacrificing Icelandic sovereignty to external interests.

This populist framing is effective precisely because it resonates with long-standing themes in Icelandic political culture. ^12^ It draws on national pride, distrust of centralised authority, and a strong belief in local control. In this sense, Euroscepticism in Iceland is not an imported ideology but an organic expression of domestic political traditions, amplified and weaponised by populist actors to challenge the status quo.

Mainstream Icelandic parties have not been immune to the appeal or pressure of Eurosceptic discourse. Their responses have varied along a spectrum from accommodation to rejection, reflecting both electoral strategies and ideological alignments.

The Independence Party has long taken an accommodating stance toward Euroscepticism. ^8^ While officially committed to Iceland’s EEA membership and greater engagement with European institutions, the party has consistently opposed moves toward full EU accession. It frames its position as a means of protecting Iceland’s sovereignty, particularly regarding fisheries and natural resources. By resonating with certain themes of populist discourse, such as the significance of national control and the dangers of bureaucratic overreach, it effectively absorbs nationalist sentiment without adopting the more extreme rhetoric of the Centre Party.

This strategy has proven electorally effective. ^22^ The Independence Party remains a significant force in Icelandic politics, partly because it can appeal to both moderate pro-Europeans and soft Eurosceptics. Its ability to navigate this line reflects the broader ambivalence in Icelandic society, where many individuals desire the benefits of European integration without the perceived costs of full membership.

In contrast, the Progressive Party has more distinctly distanced itself from hardline Euroscepticism since the departure of Sigmundur Davíð Gunnlaugsson. Aiming to rebrand itself as a centrist, pragmatic force, the party now highlights the advantages of cooperation with Europe while maintaining a cautious stance on membership. It supports the EEA agreement and Schengen participation but insists on safeguarding Iceland’s control over fisheries and agriculture.

This repositioning is both tactical and ideological. By distinguishing itself from the Centre Party, the Progressive Party can appeal to voters who are sceptical of the EU but cautious of populist extremes. It also enables the party to re-engage in constructive policymaking within coalition governments, where pragmatism often prevails over ideology.

The tangible consequences of Icelandic Euroscepticism are evident in the suspension of the EU accession process. Initiated in 2009 after the financial crisis, the process was halted in 2013 by a new right-wing government coalition that was less enthusiastic about membership. ^8^ The official explanation cited a lack of public consensus, but the deeper reason was the political importance of sovereignty. The accession talks, once seen as a pathway to recovery, started to represent a threat to Iceland’s identity and independence.

Beyond accession, Euroscepticism has had a significant impact on Iceland’s broader political culture. It has bolstered a discourse that emphasises self-reliance, democratic control, and resistance to external interference. These themes have impacted debates on energy policy, constitutional reform, foreign relations, and immigration. Even pro-European actors must frame their arguments in a manner that aligns with national sovereignty, reflecting the prevailing discourse’s dominance. ^9^

Euroscepticism in Iceland is not merely a transient phenomenon or a rhetorical device used by opportunistic populists. It is a deeply ingrained ideological stance, woven into the fabric of political identity. ^17^ Its durability lies in its capacity to connect past struggles with present concerns, portraying EU integration not solely as a policy issue but as a matter of national survival.

## Conclusion

Euroscepticism in Iceland is neither an imported phenomenon nor a recent outgrowth of the populist wave sweeping Europe. It is deeply rooted, shaped by Iceland’s unique historical experience, its successful struggle for independence, and its enduring political culture of sovereignty, self-reliance, and institutional particularism. Icelandic Euroscepticism is less concerned with migration and identity politics than its continental counterparts; instead, it is anchored in a form of economic nationalism that perceives natural resources–especially fisheries–as sacrosanct, viewing supranational governance as a direct threat to democratic control.

Quasi-populist actors, such as the Centre Party and the People’s Party, have successfully tapped into this reservoir of sentiment. They have channelled it into a narrative of elite betrayal, portraying the EU as a distant and intrusive force, and European integration as an existential challenge to Icelandic nationhood. Although coalition politics and electoral realities have constrained their impact on policy, they have nonetheless succeeded in reshaping the terms of debate, compelling mainstream parties to adopt more cautious or defensive positions on Europe.

Yet the story is not one of linear entrenchment. Recent geopolitical shocks–including the destabilising consequences of Brexit, the uneven global response to COVID-19, Russia’s renewed military aggression, and the uncertain trajectory of U.S. foreign policy under a second Trump presidency–have prompted a reevaluation in Icelandic political circles. For the first time in a decade, Europe is once again being considered as a potential strategic anchor rather than merely a bureaucratic burden.

This shift culminated in autumn 2024, when a new coalition government–comprising the Social Democratic Alliance, the Liberal Reform Party, and the previously Eurosceptic People’s Party–committed to holding a referendum on whether to resume Iceland’s EU accession process. The inclusion of this provision in the government platform marked a significant concession by the People’s Party and signalled a broader softening of Eurosceptic rigidity in the national debate.

While the referendum does not guarantee a policy change, it reflects a political and discursive opening that had remained closed since 2013. In this context, Iceland serves as a revealing case for scholars of comparative Euroscepticism. Its political dynamics illustrate the interaction of nationalism and populism in a small-state setting, where sovereignty is not an abstract principle but a lived historical imperative. The Icelandic case also highlights how Euroscepticism can evolve–not merely harden or fade–in response to shifting domestic and international pressures. As Iceland approaches another potential crossroads in its European relationship, the interplay of populism, nationalism, and strategic pragmatism will continue to define its trajectory.

## Ethics and consent

Ethical approval and consent were not required for this study, as it relies on public discourse, political party platforms, and secondary literature rather than human subjects research.

## Data Availability

No data are associated with this article. This is a theoretical and qualitative political analysis that does not rely on original quantitative datasets, images, or underlying values requiring replication.
