# Supplementary material for: EUPopLink Country report - Iceland
Source: Open Res Eur. 2026 May 7;6:153. [Version 1] doi: 10.12688/openreseurope.23658.1 (PMC13332376; doi:10.12688/openreseurope.23658.1)
Supplement: Peer review history

# Reviewer report: Reviewer response for version 1

**DOI:** 10.21956/openreseurope.25614.r74932

**Published:** 2026-07-03

**Authors:** Francesco Alicino

**Affiliations:** 1 University LUM, Bari, Italy

**Introduction**

 This report offers a clear, well-structured, and timely examination of Euroscepticism in Iceland. Its principal strength lies in situating Icelandic Euroscepticism within the country’s distinctive historical, political, and economic trajectory, rather than interpreting it as a local variant of broader European populist or anti-EU trends.

 The report argues persuasively that standard analytical categories (centred on migration, cultural identity, or generalised opposition to supranational governance) are insufficient to account for the Icelandic case. Euroscepticism here is rooted in a historically embedded concern with sovereignty, democratic self-determination, and the protection of natural resources, above all fisheries and energy sources. The report is particularly effective in tracing how these factors interact with Iceland’s domestic party system, coalition dynamics, and evolving geopolitical context, thereby offering a valuable contribution to the comparative study of Euroscepticism, nationalism, and populism in small-state settings.

**Main Comments**

 A central merit of the report is its detailed mapping of Iceland’s internal political landscape. The discussion of party positions on European integration is both informative and analytically productive. Notably, the report draws a careful distinction between the hard Euroscepticism of the Centre Party and the more welfare-oriented, reactive, and context-sensitive Euroscepticism of the People’s Party; a distinction that resists the tendency to treat populist anti-EU sentiment as a monolithic phenomenon.

 The analysis also demonstrates that Icelandic Euroscepticism is shaped by the interplay between domestic political dynamics and external developments. Unlike many other European cases, where opposition to European integration is primarily driven by identity politics or anti-elite rhetoric, the Icelandic variant appears more strongly conditioned by strategic pragmatism. Sovereignty remains a structuring concern, but it operates in conjunction with concrete policy interests: the preservation of natural resources, economic autonomy, and regulatory self-determination. Fisheries and energy sources function not merely as symbolic referents but as substantive policy domains through which anxieties about European integration are articulated and politically mobilised.

 The report is equally strong in documenting the tangible political consequences of Euroscepticism on Icelandic policymaking. The suspension of the EU accession process in 2013, the enduring influence of sovereignty discourse on debates over energy policy, constitutional reform, and immigration, and the observation that even pro-European actors are compelled to frame their arguments in terms of national sovereignty; all of these are carefully examined and constitute an important analytical contribution. Equally significant is the report’s account of the coalition government formed in late 2024, which includes the People’s Party and has committed to holding a referendum on resuming the EU accession process. This development represents a notable discursive and political shift, raising important questions about the extent to which geopolitical pragmatism can durably reshape ideological positions within a populist party. The report would benefit from engaging more explicitly with this interpretive tension.

 The report is also strong in highlighting the non-static character of Icelandic Euroscepticism. The discussion of Brexit, the COVID-19 pandemic, Russia’s war against Ukraine, and the uncertainties surrounding transatlantic security effectively illustrates how external shocks can reshape domestic political narratives. The report rightly notes that these developments have prompted a reassessment of the European Union; no longer viewed solely as an economic framework, but increasingly as a potential geopolitical and strategic anchor for a small and exposed state. This emphasis on the adaptive and evolving quality of Euroscepticism is among the most original contributions of the report.

 One area where the argument could be considerably strengthened concerns the relationship between citizens’ (and political actors’) *attitudes* towards the European Union and their actual *knowledge* of how the EU operates. This distinction is analytically crucial and, in the Icelandic case, largely unaddressed. The report references a January 2025 Prósent survey indicating that 45% of Icelandic respondents support EU membership, 35% oppose it, and 20% hold no firm position. This is a valuable empirical reference point. However, attitudinal data of this kind (measuring approval or disapproval of EU membership) does not in itself reveal whether those attitudes are grounded in an accurate understanding of the EU’s institutional architecture, its legislative procedures, the distribution of competences between EU institutions and Member States, or the mechanisms through which EU law is incorporated into domestic legal orders.

 This gap matters considerably for the report’s central argument. When Icelandic citizens express support for or opposition to EU membership, and when political parties, whether populist or mainstream, articulate pro- or anti-integration positions, it remains unclear whether these stances reflect informed assessments of what membership would concretely entail, or whether they are primarily sustained by political narratives, historical memories, and symbolic constructions of the European Union that do not always correspond to institutional reality. The two are not equivalent, and conflating them risks overstating the substantive rationality of Eurosceptic (or indeed pro-European) positions alike.

 This concern is especially pertinent given the specific nature of Icelandic Euroscepticism as described in the report. The fears most frequently articulated (over fisheries policy, energy source regulation, legal autonomy, and sovereignty) are grounded in concrete and sensitive policy domains. Yet it is an open empirical question whether Icelandic public discourse and party rhetoric accurately reflect an understanding of how EU competences actually function in these areas, how EU legislation interacts with EEA obligations already binding on Iceland, and what the practical legal differences between EEA participation and full EU membership would be. The report would benefit from asking, for instance, whether Icelandic citizens and political elites distinguish clearly between the regulatory implications of EEA membership (to which Iceland is already subject) and those of full accession; whether concerns about fisheries policy and the control of energy sources are based on accurate knowledge of the relevant EU competences and their actual scope; and whether fears about loss of sovereignty over natural resources (including fisheries and energy) reflect a precise understanding of EU institutional practice or, rather, draw on broader narratives of external interference that may not be fully consistent with institutional reality.

 Such an analysis need not transform the report into an empirical study of political knowledge. Even a more circumscribed engagement (drawing on available survey data, Eurobarometer-style research where applicable, media studies, or existing scholarship on political knowledge in small European states) would substantially enrich the work. It would allow the author to distinguish more rigorously between Euroscepticism rooted in substantive policy concerns informed by institutional knowledge and Euroscepticism driven by symbolic or narrative constructions of the EU as an existential threat to national self-determination. This distinction would not undermine the report’s core argument; on the contrary, it would deepen it by clarifying the epistemic foundations on which Icelandic Euroscepticism (in both its populist and mainstream variants) actually rests.

**Conclusion**

 The report makes a substantive contribution to the literature on Euroscepticism and populism. It offers a nuanced account of Iceland’s political landscape and demonstrates convincingly that Icelandic Euroscepticism emerges from a distinctive configuration of nationalist sentiment, sovereignty concerns, resource protectionism, and strategic pragmatism. A key analytical insight is that this Euroscepticism should not be understood as a fixed ideological position but as a flexible and responsive political phenomenon; one shaped by domestic competition, historical narratives, economic interests, and shifting international conditions.

 The report would be further strengthened by a more explicit engagement with the relationship between political attitudes and institutional knowledge. The central question is not simply whether Icelanders support or oppose EU membership, but whether their positions (and those of the political actors who mobilise them) are grounded in an accurate understanding of how the European Union actually functions: its institutional mechanisms, its legislative instruments, its relationship with Member States, and the concrete legal and policy implications of accession. Determining whether Icelandic Euroscepticism rests on such informed assessments, or whether it is primarily sustained by political narratives that do not always correspond to institutional reality, would deepen the analytical value of the report and enhance its contribution to comparative research on the relationship between political knowledge, public perception, and Eurosceptic attitudes.

If applicable, is the statistical analysis and its interpretation appropriate?

Partly

Is the study design appropriate and is the work technically sound?

Yes

Is the work clearly and accurately presented and does it engage with the current literature?

Yes

Are sufficient details of methods and analysis provided to allow replication by others?

Yes

Are all the source data and materials underlying the results available?

Yes

Reviewer Expertise:

NA

I confirm that I have read this submission and believe that I have an appropriate level of expertise to confirm that it is of an acceptable scientific standard.

## References

[1] : A blessing and a curse? Examining public preferences for differentiated integration. *European Union Politics* .2023;24(1) : 10.1177/14651165221133671 143-163 10.1177/14651165221133671

[2] : Right‐Wing Sovereignism in the European Union: Definition, Features and Implications. *JCMS: Journal of Common Market Studies* .2024;62(2) : 10.1111/jcms.13497 341-359 10.1111/jcms.13497

[3] : Cultural narrative, crisis, and contention in Iceland's bid to join the European Union, 2009–2015. *Frontiers in Political Science* .2024;5: 10.3389/fpos.2023.1017285 10.3389/fpos.2023.1017285

[4] : Iceland: Political Developments and Data in 2023. *European Journal of Political Research Political Data Yearbook* .2024;63(1) : 10.1111/2047-8852.12443 218-226 10.1111/2047-8852.12443

[5] Small States and Shelter Theory.2018; 10.4324/9780429463167 10.4324/9780429463167

[6] : Enemies Inside: European Populism and Dimensions of Euroscepticism.2024; 10.55271/pp0043b 10.55271/pp0043b

[7] : Euroscepticism as a Persistent Phenomenon*. *JCMS: Journal of Common Market Studies* .2013;51(1) : 10.1111/j.1468-5965.2012.02297.x 1-16 10.1111/j.1468-5965.2012.02297.x

[8] : Prosodic parameters for the detection of regional varieties in Italian. *https://www.researchgate.net/publication/228809041_Prosodic_parameters_for_the_detection_of_regional_varieties_in_Italian* .2007;

[9] : Euroscepticism and the Future of European Integration. *https://global.oup.com/academic/product/euroscepticism-and-the-future-of-european-integration-9780198793380* .2018;

# Reviewer report: Reviewer response for version 1

**DOI:** 10.21956/openreseurope.25614.r74931

**Published:** 2026-05-29

**Authors:** Nika Gigauri

**Affiliations:** 1 GIPA – Georgian Institute of Public Affairs, Tbilisi, Georgia

The article provides a compelling and rigorous examination of Euroscepticism in Iceland, tracing both its distinct characteristics and its historical trajectory. It offers a clear and analytically rich account of how Icelandic Euroscepticism is rooted not simply in contemporary identity politics, but in a longer national tradition centred on sovereignty, democratic control, and the protection of natural resources. The article convincingly shows that Iceland’s relationship with European integration has been shaped by a dual logic: practical participation in European frameworks, especially through the EEA and Schengen, alongside persistent resistance to full EU membership because of concerns over autonomy, fisheries, and supranational authority.

 A major strength of the article is its careful discussion of Iceland’s party-political landscape. The author provides a nuanced classification of party positions on Europe and shows that attitudes towards European integration in Iceland do not fit neatly into a conventional left-right divide. Instead, they are shaped by historical memory, sectoral interests, national identity, and sovereignty-based political narratives.

 The article also makes an important contribution by showing how Icelandic Euroscepticism has evolved in response to recent geopolitical developments, including Brexit, the COVID-19 pandemic, Russia’s war against Ukraine, and shifts in U.S. foreign policy. The discussion of the 2024 coalition government and the commitment to a referendum on resuming the EU accession process gives the article clear contemporary relevance.

 Overall, the article is clearly presented, well structured, and scientifically sound. The study design is appropriate for a country report, and the conclusions are adequately supported by the analysis.

If applicable, is the statistical analysis and its interpretation appropriate?

Not applicable

Is the study design appropriate and is the work technically sound?

Yes

Is the work clearly and accurately presented and does it engage with the current literature?

Yes

Are the conclusions drawn adequately supported by the results?

Yes

Are sufficient details of methods and analysis provided to allow replication by others?

Yes

Are all the source data and materials underlying the results available?

Yes

Reviewer Expertise:

Political Science, Party Politics, Populism, and Euroscepticism.

I confirm that I have read this submission and believe that I have an appropriate level of expertise to confirm that it is of an acceptable scientific standard.
